# A Case of Squamous Cell Carcinoma of the Renal Pelvis in association with *Schistosoma hematobium*

**DOI:** 10.1155/2012/352401

**Published:** 2012-12-19

**Authors:** Muhammad A. A. Khan, Ashok Kar, Marjorie M. Walker, Jo Lloyd, Justin A. Vale, Erik K. Mayer

**Affiliations:** ^1^Department of Urology, Imperial College Healthcare NHS Trust, St Mary's Hospital, Praed Street, London W2 1NY, UK; ^2^Department of Histopathology, Imperial College Healthcare NHS Trust, St Mary's Hospital, Praed Street, London W2 1NY, UK

## Abstract

A 72-year-old man presented with painless frank haematuria. Investigations included intravenous urogram and abdominal/pelvic CT which revealed a marked focal thickening of the wall of the inferior aspect of the left renal pelvis extending into the lower pole calyx and into the pelviureteric junction resulting in left hydronephrosis. Urine cytology demonstrated clusters of malignant keratinised squamous cells and schistosome ova. He underwent left laparoscopic radical nephroureterectomy and histology revealed moderately differentiated keratinising squamous cell carcinoma in the renal pelvis.

## 1. Introduction

In countries with a high prevalence of *Schistosoma haematobium* there is a strong association with urinary tract pathology [1]. A recognised common association is squamous cell carcinoma (SCC) of the urinary bladder [2]. Schistosomiasis involving the ureter is also associated with SCC but this is less common than bladder cancer [3]. 

In nonschistosomiasis endemic areas, SCC of the urothelium is rare and typically occurs in association with chronic inflammation associated with long-term indwelling catheters, urinary tract calculi, bladder diverticula, and chronic urinary tract infections. Only 5% of all urothelial tumours present in the upper urinary tract (ureter/renal pelvis). The Swedish Cancer Registry, Hölmang et al. [4], reports that 8% of upper urinary tract urothelial tumours are SCC, similar to previous reports of an incidence of 6–15%.

SCC of the upper urinary tract is a rare occurrence, and an association of SCC of the renal pelvis with schistosomiasis has not been previously described. We present a case of SCC of the renal pelvis which was diagnosed by urine cytology with schistosome ova alongside malignant keratinised squamous cells.

**Key Messages**

Squamous cell carcinoma of the renal pelvis is a rare complication of *Schistosoma* infection. Urine cytology is a valuable tool in the investigation of patients with haematuria and is pivotal in the diagnosis of urogenital schistosomiasis. Management depends on the extent of organ involvement but in the absence of metastatic disease, usually involving radical surgical resection with systemic treatment for schistosomiasis.

## 2. Case History

A 72-year-old man presented with two episodes of painless frank haematuria. Preceding lower urinary tract symptoms only consisted of mild hesitancy and straining with a moderate stream. There was no other significant urological history. He had smoked previously for 40 years at three packs a day. He was a submariner during the Second World War and then spent 30 years allegedly travelling in South America.

**Figure 1:**
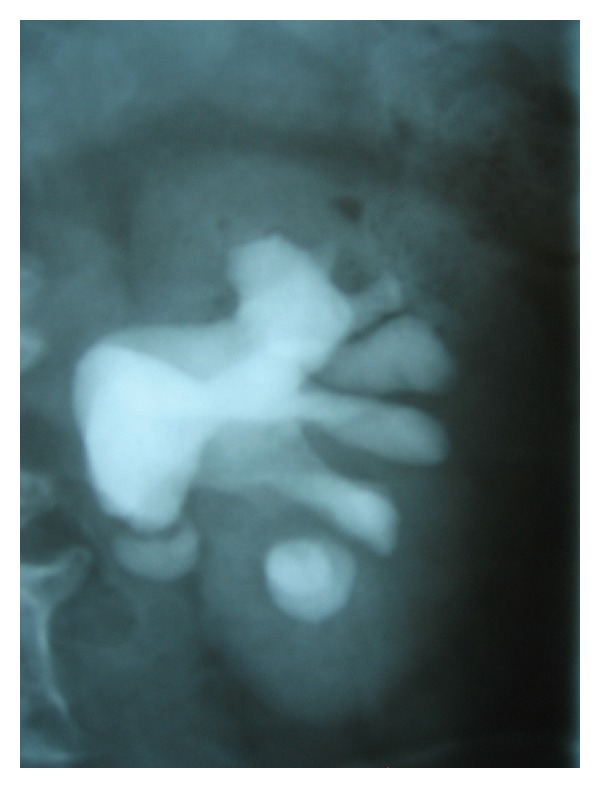
Intravenous urogram demonstrating dilatation of the left PC system with blunting and fullness of the calyces. There is an unusual crescentic shape enhancement seen inferiorly at the left renal pelvis.

**Figure 2:**
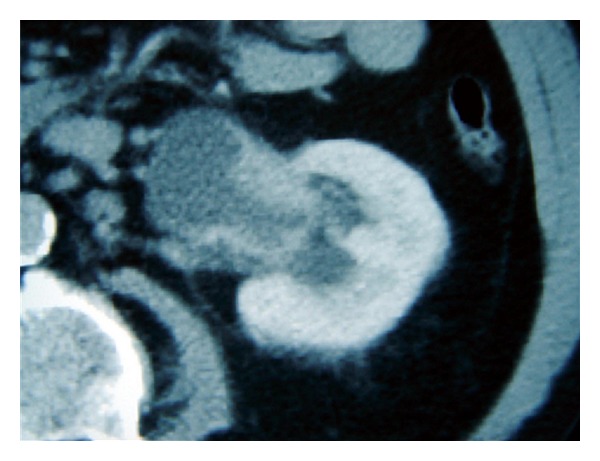
Computed tomography scan showing marked focal thickening of the wall of the inferior aspect of the left renal pelvis extending into the lower pole calyx and into the pelviureteric junction resulting in left hydronephrosis.

Ultrasound was reported as normal besides a mildly enlarged prostate. Flexible cystoscopy demonstrated a tri-lobar obstructing prostate with normal bladder urothelium. An abnormal intravenous urogram showed dilatation of the left pelvicalyceal system with blunting and fullness of the calyces. There was an unusual crescentic shape enhancement seen inferiorly at the left renal pelvis (Figure 1). A CT scan showed marked focal thickening of the wall of the inferior aspect of the left renal pelvis extending into the lower pole calyx and into the pelviureteric junction resulting in left hydronephrosis (Figure 2).

**Figure 3:**
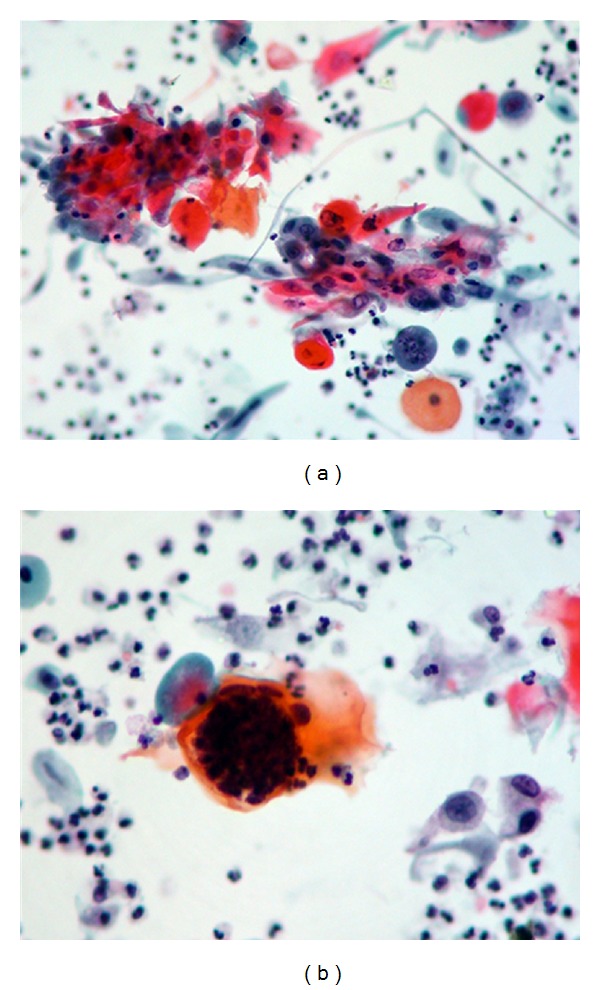
Urine cytology specimen showing (a) clusters of keratinised malignant squamous cells and (b) *Schistosoma* egg, probably with a terminally located spine.

**Figure 4:**
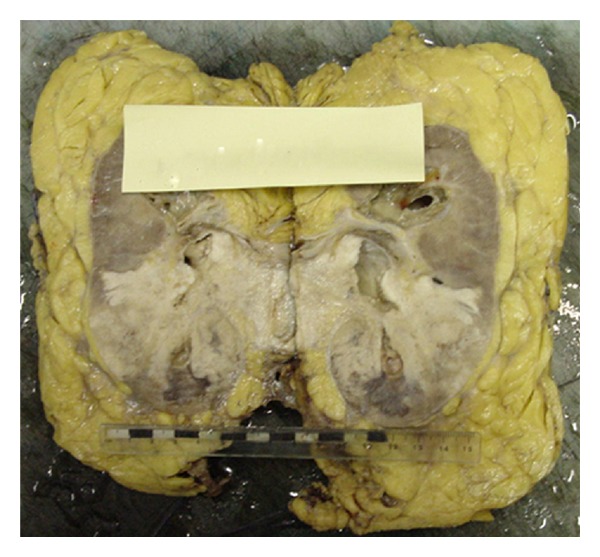
Lower pole of the left kidney largely replaced by a white friable tumour which appears to arise from the pelvicalyceal mucosa at the hilum. The tumour focally extends into hilar and perinephric fat.

There was no growth on culturing mid-stream urine. Urine cytology revealed clusters of malignant keratinised squamous cells and schistosome ova (Figures 3(a) and 3(b)). He was given praziquantel at 40 mg/kg. He underwent left laparoscopic radical nephroureterectomy without complications. Histology showed a moderately differentiated keratinising squamous cell carcinoma arising from the renal pelvis from an area of squamous metaplasia and dysplasia. The carcinoma replaced much of the lower pole of the kidney (Figure 4). The tumour focally extended into hilar and perinephric fat, with widespread lymphatic, venous, and perineural invasion. The main renal vein was not infiltrated. All 12 lymph nodes were negative for malignancy (pT4, N0, M0).

The patient presented five months later with multiple lung lesions. He declined chemotherapy and unfortunately died 10 weeks later.

## 3. Discussion

Schistosomiasis is a parasitic infection that infects 250 million people worldwide. Ten species of schistosomes can infect humans, including *S. mansoni*, *S. hematobium*, and *S. japonicum* [5]. Urinary tract disease is a hallmark of infection by *S. hematobium* due to adult schistosomes producing ova in venules of the genitourinary tract. Although involvement of urogenital organs differs markedly, it appears to correlate with the degree of venous circulation. Hence, the urinary bladder, lower ends of ureter, and seminal vesicles are most commonly affected owing to a rich venous supply [6]. 

In the upper urinary tract, ova can be found in all layers of the ureter and can lead to fibrosis and stricture formation [7]. Ureteric obstruction is therefore the most common complication of *S. hematobium* infection, particularly the endopelvic part of the ureter. Whilst SCC of the bladder is well described in the literature, SCC of the upper urinary tract in association with *S. hematobium* infection is rarely cited [3].

Squamous metaplasia is a precursor for malignant change and associated with chronic inflammation of the urothelium. Case reports of upper tract nonschistosomiasis SCC, not unsurprisingly, describe predisposing factors associated with chronic inflammation, in particular nephrolithiasis [8]. Several mechanisms are proposed to explain the association between urinary schistosomiasis and SCC of the bladder. Fibrosis induced by eggs leads to proliferation, hyperplasia, and metaplasia of the urothelium, which are precursors of malignancy [9]. Adult schistosomes and miracidia have also been shown to have raised beta glucuronidase levels and hence release potentially carcinogenic amines into the urine [10]. 

In this case, diagnosis of urinary schistosomiasis is based on the detection of *S. haematobium* ova in the urine. The ova count can be used to assess the severity of infection, and the viability of ova in any given sample has been assessed using Congo Red [5]. Novel detection techniques from urine using PCR from DNA specific fragments have also recently been reported [11]. Cystoscopy is commonly used in the evaluation of lower urinary tract lesions and diagnosis can be confirmed by finding ova with a granulomatous reaction at histology [5]. 

Praziquantel or metrifonates are systemic treatments of choice in *S. hematobium*. Praziquantel induces tetanic paralysis and decreases glucose absorption in the trematode by interfering with ionic exchange. Metrifonate is an organophosphate that also causes paralysis by irreversibly blocking cholinesterase [4]. 

Surgical management of upper tract SCC associated with schistosomiasis should follow the same principles as for any upper tract SCC. Radical nephroureterectomy is the treatment of choice for patients without metastasis and there appears to be only little benefit for neoadjuvant and adjuvant radiotherapy/chemotherapy [2]. The majority of upper tract SCCs are of an advanced stage at time of treatment (T3/T4) and this contributes to the overall poor prognosis of upper urinary tract SCC, with less than 10% survival after 5 years [2]. 

In those patients at risk for upper tract SCC, a high index of suspicion should be maintained to improve detection of disease at an earlier stage. In a case series which included nephrectomies performed for nonfunctioning kidneys secondary to staghorn calculus, the first indication of malignancy was the incidental finding of SCC on histological examination of the nephrectomy specimen [7]. This further emphasises the role of urine cytology in detecting upper tract SCC in those patients presenting with haematuria or in a population at high risk of developing SCC (e.g., chronic inflammation due to nephrolithiasis and recurrent urinary tract infections).
